# Superficially Invasive Vulvar Squamous Cell Carcinoma: A 37-Year-Long Experience of a Tertiary Referral Center

**DOI:** 10.3390/cancers13153859

**Published:** 2021-07-31

**Authors:** Mario Preti, Fulvio Borella, Niccolò Gallio, Luca Bertero, Debra Sandra Heller, Pedro Vieira-Baptista, Stefano Cosma, Federica Bevilacqua, Sebastiana Privitera, Leonardo Micheletti, Chiara Benedetto

**Affiliations:** 1 Division of Gynecology and Obstetrics 1, Department of Surgical Sciences, “City of Health and Science University Hospital”, University of Turin, 10126 Turin, Italy; mario.preti@unito.it (M.P.); niccolo.gallio@edu.unito.it (N.G.); stefano.cosma@unito.it (S.C.); federica.bevilacqua@unito.it (F.B.); leonardo.micheletti@unito.it (L.M.); chiara.benedetto@unito.it (C.B.); 2 Pathology Unit, Department of Medical Sciences, “City of Health and Science University Hospital”, University of Turin, 10126 Turin, Italy; luca.bertero@unito.it (L.B.); sprivitera@cittadellasalute.to.it (S.P.); 3 Rutgers-New Jersey Medical School, Newark, NJ 07103, USA; hellerds@njms.rutgers.edu; 4 Lower Genital Tract Unit, Centro Hospitalar de São João, 4200-319 Porto, Portugal; pedro.vieira.baptista@lusiadas.pt

**Keywords:** superficially invasive vulvar squamous cell carcinoma, vulvar cancer, perineural invasion, recurrence, groin metastases, lichen sclerosus, vulvar squamous intraepithelial lesion, human papilloma virus

## Abstract

**Simple Summary:**

Superficially invasive vulvar squamous cell carcinoma (SISCCA) is a rare subgroup of vulvar squamous cell carcinoma with good prognosis and low risk of groin metastasis, however experience reported in the literature is limited. The aim of our retrospective study is to present a large series of SISCCAs and analyze prognostic outcomes. We also described a case of SISCCA from our series with vulvar recurrence and groin metastasis, together with a literature review of this issue. Overall, SISCCA have a good prognosis, irrespective of type of surgical treatment, pathological characteristics, and status of surgical margins. However, patients should enter in accurate long term follow-up, in particular for younger patients and in case of small tumors, as recurrence or re-occurrence may occur even after many years after diagnosis.

**Abstract:**

Superficially, invasive vulvar squamous cell carcinoma (SISCCA) (FIGO stage IA) is a rare subset of vulvar cancer defined as a single lesion measuring ≤2 cm with a depth of invasion of ≤1.0 mm. This is a retrospective study performed on 48 patients with SISCCA, surgically treated between 1981 and 2018 at the S. Anna Hospital, University of Turin, to evaluate pathological characteristics and prognosis of these tumors. Ten patients (21%) recurred: seven (14%) as SISCCA and three (7%) as deeply invasive carcinoma. One case with perineural invasion and groin node metastasis at recurrence. No patient had groin lymph node metastases at initial diagnosis. Site of SISCCA, type of surgery, status of surgical margins, and histopathological features did not differ between recurrent and non-recurrent patients. We observed a non-significant trend towards an increase of recurrences in younger women (median age: 63 years vs. 70 years, *p* = 0.09), while, surprisingly, smaller tumors (<12 mm) were significantly related to tumor relapse (*p* = 0.03). Overall, SISCCA has a good long-term prognosis, regardless of the pathological characteristics and the type of surgical treatment. We recommend close follow-up, especially for younger patients and for small tumors, due to the possibility of recurrence or re-occurrence even after years.

## 1. Introduction

Vulvar cancer is a rare malignancy, accounting for 4% of all gynecological cancers in the United States of America (USA) [1]. The incidence rate is estimated to be 2.5 new cases per 100,000 women per year, and it usually occurs in postmenopausal women with a mean age at diagnosis of 68 years. The incidence rate is similar in Western Europe with 2.4 cases per 100,000 women [2].

In the previous decades, many authors [3,4,5] reported an upward trend in incidence rate in developed countries: for instance, a 20% increase from 1973 to 2000 had been observed in the USA [3]. However, more recently, a reduction in incidence trend has been reported in most Western countries, particularly among older women. In Italy, the total incidence significantly decreased over time with some differences across the decades: age-period cohort analysis revealed a risk reduction in cohorts born between 1905 and 1940 and a new increase in cohorts born since 1945 [6].

The most common vulvar cancer histotype is squamous cell carcinoma (VSCC), and it can be etiologically divided in two different entities [7,8]. The first one (warty and basaloid SCC) is associated with human papilloma virus (HPV) infection, typically affects younger women, and develops from vulvar high-grade squamous intraepithelial lesion (HSIL). The second one (keratinizing SCC) is not related to HPV infection and is thought to be an inflammation-related cancer, slowly progressing within a background of chronic inflammation usually lichen sclerosus and sometimes lichen planus. It is thought to be preceded by differentiated vulvar intraepithelial neoplasia (dVIN), although rapid progression from dVIN to SCC may be the reason dVIN is not frequently identified. Recently, non-HPV-linked VSCCs were further categorized on the basis of the presence or absence of *TP53* mutations with prognostic implication (i.e., HPV-negative patients with mutated *TP53* showed a worse outcome) [9].

Historically, Franklin and Rudledge in 1971 [10] suggested the concept of “microinvasion” in the vulva mirroring the concept of cervical microinvasion (a stromal infiltration up to 5 mm from the epithelial basement membrane) in use at that time. Unfortunately, “microinvasive” vulvar cancer showed a quite different clinical behavior from cervical cancer, and approximately 15% of patients in this category already had nodal groin metastasis. In 1983, to define a low-risk subset of vulvar cancer that could safely be treated with a conservative approach, the International Society for the Study of Vulvar Disease defined “microinvasive cancer of the vulva” (substage IA) as “a single lesion measuring 2 cm or less in diameter with a depth of invasion of 1 mm or less” [11]. This definition proposed by the ISSVD was accepted by the International Federation of Gynecology and Obstetrics (FIGO) and the American Joint Committee on Cancer (AJCC), and nowadays, SISCCA is defined as a single lesion measuring ≤2 cm, confined to the vulva or perineum and with a stromal invasion of ≤1.0 mm, without nodal metastasis (stage IA according to FIGO classification or pT1a pN0 according to AJCC-TNM system) [12,13]. The term SISCCA was put forward by the LAST Terminology Group who recommended discarding the term “microinvasive” as confusing with its varying definitions across sites. SISCCA was meant to unify terminology across sites for superficially invasive SCC, although measurements differ across genital sites [14].

Regarding the treatment for SISCCA, wide local excision or vulvectomy without groin lymphadenectomy are safe and effective therapeutic options, as the risk of nodal metastasis in stage IA is extremely low (<1%). However, few case series of SISCCA are reported in the literature, and thus available data regarding this rare entity are limited [15,16].

The aim of this study was to perform a retrospective clinicopathological and outcome analysis of a consecutive series of primary cases of SISCCA treated in a tertiary level referral center.

## 2. Methods

Patients surgically treated for SISCCA at the Department of Surgical Sciences, S. Anna Hospital, University of Turin, from 1981 to 2018 were retrospectively retrieved from an institutional database of vulvar malignant neoplasms.

The clinicopathological characteristics of patients were extracted from the medical records. They included age, tumor size (in mm), anatomical involvement (labia majora, labia minora, clitoris, fourchette), type of surgical approach (excisional biopsy, wide local excision, vulvectomy), surgical margins status (positive or negative), depth of stromal invasion (in mm), presence of perineural invasion (PNI) and lymphovascular invasion (LVI), presence of associated lesions (lichen sclerosus, lichen planus, dVIN, and HSIL), date of last follow-up visit, and recurrence.

For surgical procedures, the width of resection was about 1 cm of normal skin around the lesion, according to the site of the lesion. The depth of resection was superficial: removal of the most superficial layer with a variable amount of dermis and subcutaneous tissue [17].

Depth of invasion or stromal invasion were defined as the measurement of the tumor from the epithelial–stromal junction of the adjacent most superficial dermal papilla to the deepest point of invasion, as recommended by Wilkinson [18]. Surgical margin was considered as positive if vulvar HSIL, dVIN, or superficially invasive tumor was present at the resection margins. PNI was defined as the presence of tumor cells along nerves and/or within the epineurial, perineurial, and endoneurial spaces of the neuronal sheath [19], while LVI was defined as the presence of cancer cells inside the capillary lumens of either the lymphatic or the microvascular drainage system within the primary tumor [20]. A dedicated pathologist reassessed all the cases in order to confirm the diagnosis, to confirm the depth of invasion, and to report the presence of lymphovascular and/or perineural invasion (if not reported in the original histological examination).

All patients included in this study received regular follow-up. Follow-up visit was performed every 4 months during the first 3 years, then every 6 months for the subsequent 3 years, and then once every 12 months. An appointment for the next follow-up visit was made during each checkup. Additional clinical examination was also carried out at patient request.

The follow-up visit was performed with vulvoscopy, clinical groin examination, and biopsy in cases of any suspected lesion. Tumor recurrence was defined as any histologically confirmed recurrence of vulvar cancer. No distinction between recurrence and re-occurrence was made.

We excluded patients without histological confirmation during pathological review, or surgically treated in other institutions, or with a follow-up shorter than 6 months.

Ethics approval was not required due to the retrospective nature of the study, as stated by our institutional review board.

Statistical analyses were performed using IBM^®^ SPSS^®^ v.23 (SPSS Inc., Chicago, IL, USA) software.

Data were analyzed descriptively and represented as counts and percentages. Differences in proportions among patients who had a locoregional recurrence and those who had not were tested using Pearson’s chi-squared test or Fisher’s exact test. The analyses were conducted with a 95% confidence interval (CI), and a two-sided *p*-value of 0.05 was considered statistically significant. Due to the relatively low sample size of our SISCCA series, as well as the low number of events (i.e., recurrences), no univariate or multivariate analysis was performed.

## 3. Results

In our institution, 980 women were treated for vulvar cancer between January 1981 and January 2018. Of these, 54 were SISCCA (5.5% of the total), but we considered a total of 48 cases because six did not meet the selection criteria (two cases were not confirmed at the pathological review, while the follow-up was not available for four cases). Clinico-pathological characteristics of the study population are summarized in Table 1.

The mean age at diagnosis was 69 years (range of 42–92 years) with a mean follow-up of 92 months (range 6 months–24.8 years). The mean tumor size was 12 mm (range 4–20 mm), and labia minora were the most affected site (18/48, 37%), followed by clitoral area and labia majora (25% in both cases), and vulvar fourchette (12%). Surgical treatment consisted of wide local resection in most cases (33/48, 68%), followed by vulvectomy (21%) and excisional biopsy (10%). Surgical margins were negative in 36/48 specimens (75%), with a median tumor-free histological margin distance of 11 mm (range 5–15 mm). All the 12 patients with positive surgical margins had vulvar HSIL on resection margins, neither dVIN nor invasive carcinoma was present at margins, and no patient with positive margins underwent re-surgery. Associated vulvar chronic diseases were observed and demonstrated histologically in all cases but one: 25 lichen sclerosus, 23 HSIL, 16 dVIN, and 5 lichen planus. The 25 SISCCA associated with lichen sclerosus were also associated with dVIN in 10 cases (40%) and with HSIL in 2 cases (8%).

The mean tumor depth of invasion was 0.6 mm (range 0.01–1 mm). On histological examination, no case with LVI was observed, while in one case, the presence of PNI was reported.

No SISCCA patient had groin lymph node metastases at initial diagnosis, and no multifocal tumors were observed.

**Figure 1:**
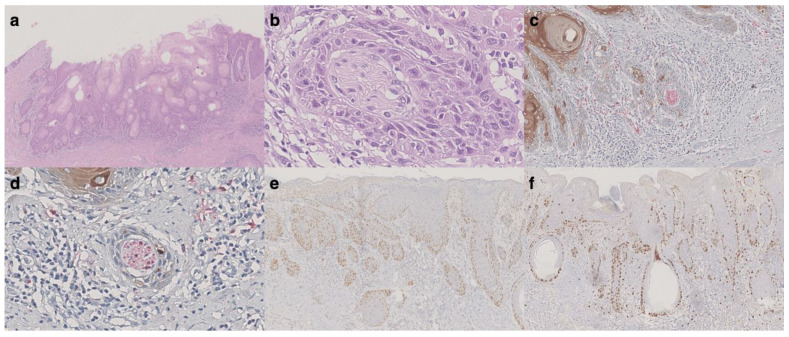
Histological features of vulvar recurrence with groin metastasis. (**a**) Hematoxylin and eosin (H&E) slide showing an invasive squamous cell carcinoma consistent with vulvar superficially invasive cancer (original magnification: 20×). (**b**) Perineural tumor invasion was observed, while no lymphovascular invasion was noted (original magnification: 400×). (**c**,**d**) Double immunohistochemical staining for S100 (red) and cytokeratins (AE1/AE3) (brown) confirming perineural invasion (original magnification for c: 200×, for d: 400×). (**e**) p53 showing widespread positivity (original magnification: 40×). (**f**) ki-67 was mostly positive in the basal layer of the non-invasive component (original magnification: 40×).

We recorded 10/48 (21%) vulvar local recurrences in our series: 7/10 cases recurred as SISCCA, while 3/10 (7%) relapsed and progressed to more invasive VSCC. The only case characterized by the presence of PNI had homolateral groin metastasis (1 positive lymph node out of 9) at the time of vulvar recurrence characterized by a SISSCA with 0.6 mm of depth of invasion (111 months after the diagnosis). The histopathological features of this case are shown in Figure 1.

The mean time to recurrence was 86.6 months (range of 8 months to 20.8 years), and only one patient died because of recurrent invasive VSCC. The mean time to recurrence as SISCCA was 50 months (5 months to 12.1 years), whereas the mean time of recurrence as more invasive VSSC was 161 months (mean 86 months to 20.8 years) (*p* = 0.038) (for details, see Table 2).

Among recurrent and non-recurrent patients, the site of SISCCA involvement, the anatomical site, the type of surgical intervention, the status of surgical margin, and the histopathological features did not differ between the two groups.

A non-significant trend towards increase of recurrences in younger women at the time of initial diagnosis (median age: 63 years vs. 70 years, *p* = 0.09) was observed, while smaller tumors were (<12 mm) significantly related to tumor relapse (*p* = 0.03).

## 4. Discussion

Our case series confirmed that vulvar SISCCA is a rare entity with good prognosis, and, according to guidelines, surgical local excision without groin lymphadenectomy is a safe and effective treatment. However, we observed a higher relapse rate (21%) compared to the two other series of SISCCA [15,16] reported in the literature (8.7% and 5.9%, respectively).

Long-term follow-up is crucial in VSCC management in order to identify recurrences. Our study shows recurrence occurring over 20 years after diagnosis. A review by Te Groothenius et al. [21] highlighted that VSCC have a recurrence rate of 4% per year, without plateauing. Furthermore, a study on the value of routine follow-up resulted in the detection of smaller recurrences in a substantial proportion of patients compared with self-reported recurrences, without a measurable effect on morbidity or mortality. It is possible that a closer follow-up in a referral center for vulvar pathology allowed for diagnosing more events.

In our series, all cases except one were associated with lesions at risk of neoplastic transformation. Lichen sclerosus is a chronic inflammatory skin disease that can lead to the development of vulvar cancer: the absolute risk of SCC in patients with vulvar lichen sclerosus varied between 0.21% and 3.88%, with increase by age, presence of VIN, a long history of lichen sclerosus, and partial compliance of treatment with topical corticosteroids [22].

Treatment with high-potency topical corticosteroids in patients with lichen sclerosus is demonstrated to reduce the risk of transformation into VSCC [23] and the risk of recurrence in lichen sclerosus-associated VSCC [24]. Corticosteroid treatment is not reported in our database of SISCCA and could have biased the assessment of risk of recurrence. The association between the presence of lichen sclerosus and VSCC recurrence is not well defined yet [21]—one study conducted on 201 consecutive VSCC reported an increased risk of vulvar local recurrence by univariate analysis hazard ratio (HR): 3.39 (95% CI 1.80–6.38) [25]; however, other authors [26,27,28] have not shown any impact on the presence of lichen sclerosus on the risk of loco-regional recurrence.

dVIN is related to non-HPV-linked vulvar oncogenesis and is frequently associated with lichen sclerosus. Bleeker et al. [29] performed a study on 3038 women affected by lichen sclerosus and reported that dVIN and vulvar lichen sclerosus were present adjacent to 25–65% of VSCC, and women with concurrent lichen sclerosus and dVIN have a 18% 10-year risk of developing VSCC compared to 2.8% in women with lichen sclerosus alone. Furthermore, Yang et al. showed a strong association between dVIN and VSCC: 58% of women with dVIN had a prior, synchronous, or subsequent invasive keratinizing VSCC [30]. This increased cancer risk has been also underlined by the higher recurrence rates (up to three times) of vulvar SCC adjacent to dVIN compared to those with nearby vulvar HSIL. Moreover, dVIN has a greater risk for malignant transformation compared to vulvar HSIL [31] with a 10-year risk of malignant transformation of 9.7% for vulvar HSIL compared to 50% of dVIN [32], and the presence of lichen sclerosus and dVIN adjacent to VSCC leads to a significantly worse prognosis [33]. It has recently been suggested that higher DNA methylation levels in VIN (both dVIN and vulvar HSIL) is related with increased disease severity and, consistent with this finding, VIN adjacent to VSCC showed a similar high-methylation pattern to VSCC [34]. The presence of lichen planus is a controversial issue—some authors suggest that in the presence of lichen planus is associated with more HSIL recurrences [35]; furthermore, in the case of coexistence with VSCC, these tumors show a more aggressive biological behavior with greater lymph node involvement, greater number of loco-regional relapses, and lower survival [36] compared with VSSC not associated with lichen planus. However, other authors have reported no association between lichen planus and VSCC [37].

Focusing on SISCCA, Grimm et al. [15] reported the presence of lichen sclerosus and dVIN in all four reported recurrences. Similarly, in our series, dVIN was found in all patients who relapsed, but no significant differences were found with non-recurrent patients. Moreover, we did not find any significant difference when considering the other associated lesions (lichen planus and HSIL). The role of these lesions in the risk of recurrence in SISCCA is therefore not yet defined.

The main treatment for SISCCA in our institution consisted in complete resection of the tumor, without groin lymphadenectomy. Usually, wide local resection was enough to obtain negative surgical margins. Some cases with an extensive preneoplastic lesion have been treated with simple vulvectomy with the removal of the skin along with the superficial portion of the fatty tissue (hypodermal) above the superficial fascia, while in a few selected cases with small lesions, an excisional biopsy was considered therapeutic. In agreement with literature data [21], we found no significant differences in recurrence rate by surgery type or surgical margin status. In particular, no patient of our series had invasive tumor at resection margins. A recent study [38] on 112 VSCCs (including 54 FIGO stage I, of which 7 were IA and 43 were IB) demonstrated a potential benefit in re-intervention if tumor-free margin was <3 mm. Other authors have also shown an increased risk of recurrence, particularly if the tumor-free surgical margin is <5 mm [39,40]; however, the prognostic significance of surgical margins status in SISCCA is still unclear. There is no evidence that a more aggressive surgical approach provides a benefit in disease free-survival (DFS) and overall survival (OS). Our study further confirms how surgery in these cases can be modulated according to the patient’s characteristics.

No patient with SISCCA had lymph node metastases at initial diagnosis in our series, thus confirming that routine lymph node assessment is not recommended. However, by assessing our database of vulvar cancer patients’ cases with depth of invasion from 1.1 to 2 mm and tumor size ≤2 cm, we found 3 cases of groin involvement out of 15 cases (20%). This finding confirms that, if the limit of 1 mm of depth of invasion is exceeded even slightly, the surgical approach must also consider groin lymph node evaluation (sentinel node biopsy +/− groin lymphadenectomy).

We found a non-significant trend for a greater number of recurrences in younger women; however, age at diagnosis is another controversial issue: a study reported an increased, although not significantly, risk in patients over 75 years old at diagnosis (HR 1.93, 95% CI 0.92–4.07) [25], while another one reported a decreased risk over 75 years (HR 0.35, 95% CI 0.17–0.73) [27].

Surprisingly, we observed a significantly higher rate of relapses in patients with a tumor extension (median diameter) less than 12 mm. A possible explanation for this finding could be that these small tumors have microscopic satellite lesions not evident on clinical examination, and multifocality is a possible risk factor for recurrence [41,42]. Multifocality for vulvar cancer is reported as an independent risk factor for poorer disease-free survival after surgery: relative risk (RR) = 3.36 [41].

Three cases in our series relapsed as deep invasive VSCC (depth of invasion >1 mm), and despite the low number of events, these recurrences showed a much longer time to relapse than cases that recurred as SISCCA. Therefore, these cases likely represent new cancers rather than recurrences, and, given the extended disease-free interval before the onset of the relapse, it is important to underline again the importance of adequate follow-up, even years after the diagnosis of the first tumor and the topical steroids treatment in case of underlying lichen sclerosus.

Finally, we reported a new case of vulvar SISCCA recurrence associated with nodal metastasis and, interestingly, the tumor was the only case in the literature showing PNI.

The risk of nodal recurrence is very low in SISCCAs—to date, only 12 cases of SISCCA with regional lymph node recurrence have been reported [15,43,44,45,46,47,48,49,50,51]. There is no specific histopathological pattern predicting nodal involvement and thus allowing tailored management. In Table 3, we review the literature and provide the cases of SISCCA that developed groin metastases.

The mean age of the previously published 12 patients was 62 years (range of 39–84 years). Depth of invasion was reported in 9 cases: mean 0.71 mm, range 0.08–1 mm. No LVI or PNI was reported in any of the cases. Associated lichen sclerosus was present in seven cases and vulvar SIL in another seven patients (six HSIL and one low-grade SIL). All groin metastases were homolateral to the primary lesion.

Some points should be underlined: the case reported by Thangavelu [47] cannot be considered a FIGO stage IA VSCC because it was not unifocal at presentation. Volgger’s [46] SISCCA was diagnosed in an immunosuppressed kidney transplant recipient and the time to groin recurrence (4 months) is short compared to other reported cases, suggesting a possible promoting role of iatrogenic immunosuppression. Sidor [48] detailed a maximum depth of invasion of 0.08 mm, along with an exophytic tumor thickness of 5 mm. Some authors suggest that exophytic growth could be a surrogate of depth of invasion [52], which suggests this case could not truly fit the SISCCA criteria. Schausberger [50] reported a SISCCA associated with an extensive and multifocal VIN 3 (vulvar HSIL) treated with a combined surgical approach and laser vaporization, which may have hampered depth of invasion assessment. Finally, in two cases, SISCCA was only found in preoperative biopsies while the surgical specimen was negative [49,51].

PNI is a pathological feature that is not taken into account by FIGO staging, although the pathologist commonly reports it if present [53]. PNI has been associated with tumor recurrence and worse outcomes in many solid tumors including prostate, head and neck, colorectal, and gastric cancer [19]. Increasing evidence suggests PNI is also an independent prognostic factor for VSCC. Patients with PNI have shorter progression-free survival and overall survival. A recent study demonstrated that PNI-negative compared with PNI-positive early stage VSCC (stage I–II) are associated with longer DFS (76% vs. 25% at 5 years, *p* = 0.05) and longer OS (92% vs. 60% at 5 years, *p* = 0.01) [54]. Role of PNI in lymph node spread is still unknown, and it is hypothesized that the nerve sheath may act as a reservoir for cancer cells that move towards the regional draining lymph node [19]. Cancer cells could proliferate in this privileged microenvironment that may facilitate pro-invasive signaling. Our case is anecdotal, but the presence of PNI may suggest a more aggressive behavior of SISCCA.

The strengths of this study include the large cohort of analyzed patients with long-term follow-up, the completeness of the clinical data, and the review of histologic slides performed by an expert pathologist in this field (SP). All cases were surgically treated by two expert gynecologists in the management of vulvar cancer diseases (L.M. and M.P.). The main limitations of this study are related to its retrospective and monocentric nature, the relatively low number of patients with SISSCA, and the potential differences in terms of patients’ management, which was modulated accordingly to their characteristics.

## 5. Conclusions

With our study, we have confirmed that vulvar SISCCA is a tumor characterized by an overall good prognosis. We confirm that the choice of surgery type and the status of surgical margins do not seem to be associated with the risk of recurrence, nor does the concomitant presence of pre-neoplastic lesions. On the basis of these data, we advise the tailoring of treatment according to patients’ characteristics, and groin surgery can be safely omitted. However, if LVI and/or PNI are observed on preoperative biopsy histological examination, sentinel lymph node biopsy may be considered.

We also report a SISCCA with metastatic lymph nodal groin recurrence. However, this pattern of recurrence remains extremely rare for vulvar SISCCA and even the few cases reported in the literature have some pitfalls.

Finally, we recommend a close and long-term follow-up, especially for younger patients and in case of small tumors, due to the possibility of recurrence or the appearance of a new, deepest invasive tumor even after many years.

## Figures and Tables

**Table 1:** Clinico-pathological characteristics of the analyzed SISCCA series.

Category		All Patients *n* = 48	No Recurrence *n* = 38	Loco-Regional Recurrence *n* = 10	*p*-Value
Mean age at diagnosis (range)		69 (42–92)	70 (43–70)	63 (42–80)	0.09
Age ≤69 years	Yes	21 (44%)	15 (39%)	6 (60%)	0.16
	No	27 (56%)	23 (61%)	4 (40%)	
Mean tumor size (mm) (range)		12 (6–20)	12 (6–20)	10 (4–19)	0.28
Tumor size ≤12 mm	Yes	29 (60%)	20 (53%)	9 (90%)	0.03
	No	19 (40%)	18 (47%)	1 (10%)	
**Site of SISSCA Involvement**					
Labia majora	Yes	12 (25%)	10 (26%)	2 (20%)	0.17
	No	36 (75%)	28 (74%)	8 (80%)	
Labia minora	Yes	18 (38%)	14 (37%)	4 (40%)	0.85
	No	30 (62%)	24 (63%)	6 (60%)	
Clitoris	Yes	12 (25%)	10 (26%)	2 (20%)	0.16
	No	36 (75%)	28 (74%)	8 (80%)	
Fourchette	Yes	6 (13%)	4 (11%)	2 (33%)	0.64
	No	42 (87%)	34 (89%)	8 (77%)	
**Surgical Treatment**					
Excisional biopsy		5 (10%)	3 (8%)	2 (20%)	0.53
Wide local excision		33 (69%)	27 (71%)	6 (60%)	
Vulvectomy		10 (21%)	8 (21%)	2 (20%)	
**Surgical Margin Status**					
Positive		12 (25%)	10 (26%)	2 (20%)	0.51
Negative		36 (75%)	28 (74%)	8 (80%)	
**Histological Features**					
Mean depth of invasion (mm) (range)		0.6 (0.01–1)	0.6 (0.01–1)	0.5 (0.2–1)	0.41
Depth of invasion ≤0.6 mm	Yes	27 (56%)	20 (53%)	7 (70%)	0.27
	No	21 (44%)	18 (47%)	3 (30%)	
Lichen sclerosus	Yes	25 (52%)	21 (55%)	4 (40%)	0.30
	No	23 (48%)	17 (45%)	6 (60%)	
Lichen planus	Yes	5 (10%)	4 (11%)	1 (10%)	0.96
	No	43 (90%)	34 (89%)	9 (90%)	
dVIN	Yes	16 (33%)	14 (37%)	2 (20%)	0.46
	No	32 (77%)	24 (63%)	8 (80%)	
VHSIL	Yes	23 (48%)	17 (45%)	6 (60%)	0.69
	No	25 (52%)	21 (55%)	4 (40%)	
Lichen sclerosus + dVIN or HSIL	Yes	12 (25%)	10 (26%)	2 (20%)	0.68
	No	36 (75%)	28 (74%)	8 (80%)	
LVI	Yes	0 (0%)	0 (0%)	0 (%)	NP
	No	48 (100%)	38 (100%)	10 (100%)	
PNI	Yes	1 (2%)	0 (0%)	1 (10%)	NP
	No	47 (98%)	38 (100%	9 (90%)	

SISCCA: superficially invasive squamous cell carcinoma; dVIN: differentiated vulvar intraepithelial neoplasia; VHSIL: vulvar high-grade squamous intraepithelial lesion; LVI: lymphovascular invasion; PNI: perineural invasion; NP: not performed.

**Table 2:** Characteristics of patients with recurrent SISCCA or invasive vulvar cancer.

*N*. Case	Age at Diagnosis (years)	Tumor Size (mm)	Surgical Treatment	Margin Status (mm)	Depth of Invasion (mm)	Associated Lesions	LVI	PNI	Type of Recurrence	Time to Recurrence (Months)
1	60	10	Excisional biopsy	Negative (8 mm)	0.5	LS	No	No	SISCCA	8
2	73	10	Wide local excision	Negative (9 mm)	1	LS	No	No	Invasive VSCC	147
3	74	10	Vulvectomy	Negative (6 mm)	0.6	LS	No	Si	SISCCA	8
4	46	20	Wide local excision	Positive	0.3	H-SIL	No	No	SISCCA + inguinal lymphnode involvement	111
5	58	5	Wide local excision	Negative (6 mm)	0.5	LS + H-SIL	No	No	Invasive VSCC	249
6	78	10	Wide local excision	Positive	0.8	H-SIL	No	No	SISCCA	5
7	80	10	Excisional biopsy	Negative (11 mm)	0.2	LS + dVIN	No	No	SISCCA	15
8	42	6	Vulvectomy	Negative (20 mm)	0.2	H-SIL	No	No	SISCCA	145
9	66	10	Wide local excision	Negative (10 mm)	1	LS	No	No	Invasive VSCC	86
10	48	10	Wide local excision	Negative (10 mm)	0.2	LS + dVIN	No	No	SISCCA	61

LVI: lymphovascular invasion; PNI: perineural invasion SISCCA: superficially invasive squamous cell carcinoma; LS: Lichen sclerosus; dVIN: differentiated vulvar intraepithelial neoplasia; H-SIL: high-grade squamous intraepithelial lesion; VSCC: vulvar squamous cell carcinoma.

**Table 3:** Summary of histopathologic and outcome features of reported vulvar SISCCA with groin recurrences.

Author	Age at FS (years)	Depth of Invasion (mm)	Size of Lesion (cm)	Associated Diseases	Surgical Treatment	Time to Recurrence (Months)	Treatment of Recurrence	Positive LNs	Survival after FS (Months)	LVI	PNI
Atamdede et al., 1989 [43]	75	0.72	1	Diffuse vulvitis, dystrophic changes of the vulva	WLE	14	Bilateral inguinal and deep pelvic LAE + RT	All left-sided LNs	-	NO	-
Van Der Velden et al., 1992 [44]	84	0.3	0.8	-	Excisional biopsy and two LN biopsy	18	Left inguinal LAE + RT	Left LNs (ECG)	24	NO	-
Hicks et al., 1993 [45]	66	0.5	-	LS	Radical WLE	12	Left inguinal femoral LAE + RT	Superficial inguinal LNs	-	NO	-
Volgger et al., 1997 [46]	39	<1	0.3	Immunosuppressive treatment and VIN 3	Simple vulvectomy	4	Bilateral inguinal-femoral LAE	2 right LNs (one with ECG)	20	NO	-
Thangavelu et al., 2006 [47]	64	<1	-	VIN 1, LS	Vulvectomy with clitoral sparing	3	Right superficial and deep LAE + RT	7 right LNs (ECG)	4	NO	-
Thangavelu et al., 2006 [47]	51	0.9	-	VIN 3, LS	Vulvectomy	36	Block dissection right groin LAE + RT	1 right groin LN	44	NO	-
Sidor et al., 2006 [48]	80	0.08	1.7 × 1.5	-	Anterior radical vulvectomy	21	Bilateral inguinal LAE + RT	1 left inguinal LN	98	NO	-
Vernooij, 2007 [49]	45	1 (biopsy only)	-	VIN 3, LS	Local radical excision	24	Left inguinal LAE + RT	1 left LN (ECG)	168	NO	NO
Vernooij, 2007 [49]	80	1	1 × 0.7	VIN 2, LS	Partial vulvectomy	13	Left inguinal LAE + RT	2 left LN (ECG)	-	NO	-
Schausberger et al., 2007 [50]	53	<1	-	VIN 3, CIN 3	Excision and laser therapy	24	Right node excision	1 right LN	36	-	-
Iyibozkurt et al., 2010 [51]	62	1 (biopsy only)	-	-	Local radical excision + bilateral inguinal LAE	27	Biopsy right groin LN	1 right LN	31	-	-
Grimm et al., 2019 [15]	44	0.9	0.3	VIN 3, LS	Anterior vulvectomy	17	Bilateral inguino femoral LAE	1 right LN	29	-	-
Present study, 2021	73	0.6	0.5	dVIN, LS	Wide local excision	8	Left inguinal LAE + WLE + RT	1 left LN	67	NO	YES

FS: first surgery; LVI: lymphovascular invasion; PNI: perineural invasion; WLE: wide local excision; LN: lymph node; LS: lichen sclerosus; LAE: lymphadenectomy; RT: radiotherapy; VIN: vulvar intraepithelial neoplasia; dVIN: differentiated vulvar intraepithelial neoplasia; CIN; cervical intraepithelial neoplasia; ECG: extracapsular growth.

## Data Availability

Data are encrypted and are available on cloud upon reasonable request from the authors.
